# Concurrent Chemoradiation for Cancer of the Cervix: Results of a Multi-Institutional Study From the Setting of a Developing Country (India)

**DOI:** 10.1200/JGO.2015.000877

**Published:** 2015-09-23

**Authors:** Ambakumar Nandakumar, Goura Kishor Rath, Amal Chandra Kataki, P. Poonamalle Bapsy, Prakash C. Gupta, Paleth Gangadharan, Ramesh C. Mahajan, Manas Nath Bandyopadhyay, Kumaraswamy, Elizabeth Vallikad, Rudrapatna N. Visweswara, Francis Selvaraj Roselind, Krishnan Sathishkumar, Dampilla Daniel Vijaykumar, Ankush Jain, Kondalli Lakshminarayana Sudarshan

**Affiliations:** **Ambakumar Nandakumar, Francis Selvaraj Roselind, Krishan Sathishkumar, Dampilla Daniel Vijaykumar, Ankush Jain**, and **Kondalli Lakshminarayana Sudarshan**, National Centre for Disease Informatics and Research; **P. Poonamalle Bapsy**, Apollo Hospitals; **Kumara Swamy**, HealthCare Global-Bangalore Institute of Oncology; **Elizabeth Vallikad**, St. John's Medical College; **Rudrapatna N. Visweswara**, International Medical School-M.S. Ramaiah Medical College, Bangalore; **Goura Kishor Rath**, Institute Rotary Cancer Hospital, All India Institute of Medical Sciences, New Delhi; **Amal Chandra Kataki**, Dr. B.B. Borooah Cancer Institute, Guwahati; **Prakash C. Gupta**, Healis-Sekhsaria Institute of Public Health, Navi Mumbai; **Paleth Gangadharan**, Amrita Institute of Medical Sciences and Research Centre, Kochi; **Ramesh C. Mahajan**, Post Graduate Institute of Medical Education and Research, Chandigarh; and **Manas Nath Bandyopadhyay**, Cancer Centre Welfare Home and Research Institute, Kolkata, India.

## Abstract

**Purpose:**

The primary output of hospital-based cancer registries is data on cancer stage and treatment-based survival that can be used to evaluate patient care, but because there are many challenges in obtaining follow-up details, a separate study on patterns of care and patterns of survival for patients at selected sites was initiated under the National Cancer Registry Programme of India. This article presents the results for cervical cancer.

**Patients and Methods:**

A standardized patient information form was used to record patient information, and data were entered into a central repository—the National Centre for Disease Informatics and Research. The study patients were from 12 institutions and were diagnosed between January 1, 2006, and December 31, 2008. Patterns of treatment were assessed for 7,336 patients, and patterns of survival were determined for 2,669 patients from six institutions, at least 70% of whom had data regarding follow-up as of December 31, 2012.

**Results:**

Of 7,336 patients, 55.5% received optimal radiotherapy (RT). In all, 80.9% of patients had locally advanced cancers (stage IIB to IVA), 51.1% received RT alone, and 44.4% received concurrent chemoradiation (RTCT). In 1,753 patients with locally advanced cancers, significantly better survival was observed with RTCT than with RT alone (5-year cumulative survival, 70.2% *v* 47.3%; hazard ratio, 0.48; 95% CI, 0.41 to 0.56).

**Conclusion:**

A conservative estimate indicates that, on an annual basis, 38,771 patients with cervical cancers in India alone do not get the benefit of RTCT and thus they have poorer survival. There is a need to reiterate the National Cancer Institute's alert that advised supplementing chemotherapy to radiation for locally advanced cancer of the cervix in the context of the developing world, where 84.3% of cancers of the cervix occur.

## INTRODUCTION

Cervical cancer comprises 7.92% of cancers in women worldwide,^1^ and in India alone, estimates indicate that there will be approximately 94,000 new cases per year.^2^ Information on care for patients with cancer and patient survival is essential in assessing cancer treatment services, and a hospital-based cancer registry (HBCR) is central to this effort.^3^ In developing countries, follow-up after treatment presents many challenges.^4^ The main aim of this study on patterns of cancer care and survival (POCCS) was to obtain information on treatment based on clinical stage and on survival for patients with cancers of the cervix, breast, and head and neck. This study of POCCS presents results on cervical cancer.

The broad concept of POCCS in cancer of the cervix is not new.^5–10^ Previous publications from India^11–14^ are from individual hospitals. Here, we present findings from pooled multi-institutional data.

An enhanced version of the prior technique^15^ for capturing electronic data and using the Internet to transmit that data to a central repository constituted the basic design and framework on which the required clinical information was obtained.

## PATIENTS AND METHODS

Twelve institutions (centers) participated in the study. The names of the institutions along with members of the Patterns of Cancer Care and Survival Group are provided in the Appendix (online only). A standardized patient information form (Data Supplement) created by oncologists with specific expertise in treating cancer of the cervix was hosted on the Hospital-Based Cancer Registries Web site. A printed form with instructions was supplied for each study participant. Trained staff completed the form by using patient and/or attendant interviews, by scrutinizing medical records and other relevant documents and registers, and by having discussions with concerned clinicians. Collaborating centers were given individual login IDs and passwords along with instructions for entering data online to be electronically transmitted to a central repository—the National Centre for Disease Informatics and Research (NCDIR). The mandate and mission statements of this one-of-a-kind center (an outcome of the National Cancer Registry Programme of the Indian Council of Medical Research) are provided at the NCDIR Web site.

### Selection Criteria

Treatment patterns based on cancer stage were examined for 7,336 newly diagnosed (January 1, 2006, to December 31, 2008) patients with cervical cancer treated at their respective institutions. However, survival analysis was restricted to data from six centers that had follow-up information for at least 70% of their respective patients as of December 31, 2012. The total number of patients was 2,686, but 17 had no details on follow-up after the date of last treatment; therefore, they were excluded, leaving 2,669 patients. Some institutions had details on follow-up for more than 70% of their patients; thus, the overall pooled percentage of patients with follow-up information for survival analysis was 87%.

The main end point, overall survival, was defined as date of diagnosis to date of death from any cause (when death was before January 1, 2013). All other patients were regarded as alive, and the last date of follow-up was the censored date. The number and proportion of patients with toxicity (in both early and late complications) and recurrence are based on any one such reported event.

Only squamous cell carcinomas are included. All centers followed the International Federation of Gynecology and Obstetrics (FIGO) staging system.^16^ Detailed survival analysis and discussion are focused on locally advanced cervical cancer (FIGO stage IIB to IVA) because advances in treatment (especially concurrent chemoradiation [RTCT]) relate to this category, which involved 72.3% of the patients (ie, 1,930 of 2,669).

### Radiotherapy

The standard practice of administering approximately 50 Gy (total dose in 20 to 25 fractions) radiation to the entire pelvis was followed.^17,18^ Other parameters such as the use of intracavity brachytherapy, use of a radiotherapy (RT) machine (linear accelerator or cobalt-60), number of fields, and duration/fractionation of RT were also considered. To simplify analysis, the term “optimal radiotherapy,” as outlined by the Chemotherapy for Cervical Cancer Meta-Analysis Collaboration^19^ and Shrivastava et al^18^ was used for this study. Optimal RT is defined as administering at least 45 Gy by external beam (minimum of 20 fractions) plus intracavity brachytherapy (any dose). All other types of RT were classified as suboptimal. The RT machine used and beam arrangement (fields) were separately factored and were adjusted for in the statistical analysis. Only a few patients received RT via intensity-modulated RT, image-guided RT, or other types of RT, and these factors were disregarded.

### Chemotherapy

Chemotherapy (CT) was administered within 1 week before or 1 week after the start of RT in 89.1% of the patients. The predominant protocol was monotherapy with cisplatin (cis-dichlorodiammine platinum). Patients who received other drugs alone or in combination with cisplatin were grouped separately. The total dose of cisplatin, the number of cycles, and dose in mg/m^2^ per cycle were calculated. However, to simplify analysis, we used only the total dose of cisplatin given. Dose was grouped a priori into less than 150 mg, 150 to 199 mg, 200 to 239 mg, and ≥ 240 mg. The mean and median number of cycles was four, and 66.2% of patients received four or more cycles. The average computed dose per cycle was 40 mg/m^2^ per week. More than 75% of patients received at least 39 mg/m^2^ cisplatin per week. The median total dose of cisplatin was 200 mg, and the average number of weeks of administration was 3.9.

### Software Applications and Quality Checks

In-house Internet-based software applications on the Hospital-Based Cancer Registry and NCDIR Web sites were modeled for data capture, checking at data provided at entry and, subsequently, tracking patient follow-up, updating treatment information, and recording follow-up details. Collaborating centers were provided exclusive login IDs and passwords with instructions for data entry and transmission. The data were downloaded periodically at the NCDIR. Data checks included checking dates and verifying discrepancies in clinical information (Data Supplement). Lists of incorrect or unlikely cases were sent to the appropriate centers for rectification. In addition, a center-wise random sample of 10% of the cases was created, and centers were asked to re-abstract the medical records for certain essential parameters.

### Statistical Analysis

Kaplan and Meier^20^ and Cox proportional hazards ratio ^21^ analyses in the SPSS software package (SPSS, Chicago, IL) were used to calculate the 5-year cumulative survival (FCS) percentages and fatality risk (with statistical significance), respectively. Multivariable analysis was performed by using Cox proportional hazards regression analysis.

## RESULTS

Table 1 provides patient, diagnostic, and treatment characteristics for 7,336 patients with cervical cancer for whom patterns of care (POC) were examined and for the 2,669 patients for whom patterns of survival (POS) were analyzed.

**Table 1:** Patient, Diagnostic, and Treatment Characteristics in Patients With Cervical Cancer Examined for POC and POS

Characteristic	POC(n = 7,336)		POS(n = 2,669)	
	No.	%	No.	%
Patient				
Median age, years	50		51	
Performance status ≥ 50%				
Before CDT		92.5		84.0
After 6 to 12 weeks of CDT		73.2		75.7
Waiting time less than 1 month				
Hospital registration and diagnosis		94.6		95.4
Diagnosis and start of CDT		65.9		69.0
Hemogram performed (including percentage of Hb)		88.5		89.5
Diagnostic				
Histologic subtype of squamous cell cancer				
Keratinizing		14.9		26.0
Nonkeratinizing large cell		27.3		49.3
Other		57.8		24.7
Tumor grade				
Well differentiated		1.2		0.7
Moderately differentiated		14.4		18.4
Poorly differentiated		25.5		33.2
Unspecified		59.0		47.7
Assessment of stage				
One consultant oncologist		43.3		44.1
Two consultant oncologists		29.1		51.9
FIGO stage proportions				
I		10.7		13.9
II		38.1		48.0
III		46.3		33.9
IV		4.6		3.9
FIGO regrouped stage proportions				
IA		0.8		0.4
IB-IIA (early stage)		15.7		23.7
IIB-IVA (locally advanced)		80.9		72.3
IVB		2.3		3.2
Treatment				
CDT with curative intent		90.1		94.2
Treatment time, days				
Mean	71		78	
Median	56		61	
Completed initial CDT within 3 months		86.6		87.1
Received optimal RT		55.5		56.3
Teletherapy plus brachytherapy		70.7		81.3
Received cisplatin		90.7		95.6
Received at least 150 mg cisplatin		61.0		69.7
Patients with early-stage (IB-IIA) cancer	1,153		632	
Received RT only		31.8		43.8
Received RTCT		30.6		38.8
Any surgery with or without RT, CT, or RTCT		36.8		17.1
Patients with locally advanced (IIB-IVA) cancer	5,933		1,930	
Received RT only		51.1		47.1
Received RTCT		44.4		50.9
Other combinations		4.5		2.0

NOTE. Proportions may not total 100% because of unknowns. There were fewer patients with stage IA and IVB to provide proportions of types of treatment; therefore, they were not included.

Abbreviations: CDT, cancer-directed therapy; CT, chemotherapy; FIGO, International Federation of Gynecology and Obstetrics; Hb, hemoglobin; POC, patterns of care; POS, patterns of survival; RT, radiotherapy; RTCT, concurrent chemoradiation.

POC was analyzed for 7,336 patients of whom 55.5% received optimal RT. Among CT recipients, 90.7% received cisplatin as a single drug, and 61% of these received a total dose of at least 150 mg of cisplatin. In all, 80.9% of patients had locally advanced cancers (stage IIB to IVA), 51.1% received RT alone, 44.4% received RTCT, and 4.5% received other combinations of treatment.

POS was analyzed for 2,669 patients; there were only 12 patients with stage IA and 85 with stage IVB, and survival was not examined. For 632 patients with early-stage cancers (stage IB to IIA; FCS, 78.4%), surgery alone or surgery with RT and/or CT significantly benefitted survival (FCS, 91.2%; hazard ratio [HR], 0.33; 95% CI, 0.17 to 0.63) compared with RT alone. In patients with early-stage cancer, no statistically significant difference in survival was observed between those who received RTCT or RT alone (FCS, 78.5% *v* 73.6%; HR, 0.79; 95% CI, 0.55 to 1.12).

### Survival in Locally Advanced (stage IIB-IVA) Cervical Cancer

Overall, in 1,930 patients, those who received RTCT had significantly better FCS compared with those who received RT alone (FCS, 70.3% *v* 43.6%; HR, 0.43; 95% CI, 0.37 to 0.49). Of the 1,930 patients, 39 were treated with other combinations of cancer-directed therapy and 138 had palliative or incomplete RT. Further comparisons and survival analysis between the RT and RTCT groups was therefore restricted to 1,753 patients (RT, 808; RTCT, 945). We saw significantly better survival with RTCT (FCS, 70.2% *v* 47.3%; HR, 0.48; 95% CI, 0.41 to 0.56) and also when cisplatin was administered as a single drug (in 903 of 945 patients who received RTCT) with RT (FCS, 70.7% *v* 47.3%; HR, 0.47; 95% CI, 0.40 to 0.55).

Patient, diagnostic, and treatment characteristics in the RT and RTCT groups are compared in Table 2. The relative proportions in the RT parameters such as teletherapy dose, teletherapy plus brachytherapy combination, number of RT fractions, deciphered optimal RT (based on these three factors), type of RT machine, and RT fields suggest that those who received concurrent CT received better delivery of RT. In the first instance in Table 3, optimal RT was adjusted for RT machine and RT field; only RT field remained significant. Data in Table 3 show that patients who received cisplatin at 150 mg or more had better survival, although there was no survival difference in subgroups who received a total dose of more than 150 mg. Patient data for those who received optimal RT were adjusted compared with data for those who received a total dose of at least 150 mg cisplatin.

**Table 2:** Comparison of Patient, Diagnostic, and Treatment Characteristics for Patients With Locally Advanced (stage IIB-IVA) Cervical Cancer Between Patients Who Received RT Alone and Those Who Received RTCT

Characteristic	RT(n = 808)		RTCT(n = 945)	
	No.	%	No.	%
Patient				
Median age, years	54		50	
Performance status ≥ 50%				
Before CDT		77.0		90.0
After 6 to 12 weeks of CDT		66.5		85.1
Waiting time of less than 1 month				
Hospital registration and diagnosis		95.3		95.7
Diagnosis and start of CDT		73.1		68.9
Follow-up proportion		86.4		87.7
Hemogram performed (including percentage of Hb)		90.0		92.1
Diagnostic				
Histology subtype of squamous cell cancer				
Keratinizing		24.4		28.9
Nonkeratinizing large cell		54.7		48.7
Other		20.9		22.4
Tumor grade				
Well differentiated		0.5		0.7
Moderately differentiated		16.1		23.3
Poorly differentiated		36.6		34.6
Unspecified		46.8		41.4
Assessment of stage				
One consultant oncologist		44.8		38.9
Two consultant oncologists		53.0		56.6
Treatment				
Treatment time, days				
Mean	67		78	
Median	58		63	
Completed initial CDT within 3 months		89.5		87.0
Details of RT				
Teletherapy dose ≥ 45 Gy		74.3		79.4
Fractions ≥ 20		87.8		98.4
Teletherapy plus brachytherapy		78.1		92.3
Received optimal RT		59.5		72.3
Linear accelerator		64.5		73.4
Four or more fields		68.8		88.9
Early and/or late complications		19.1		23.9
Recurrence		13.7		13.2
Died*		56.5		66.7

NOTE. Proportions may not total 100% because of unknowns.

Abbreviations: CDT, cancer-directed therapy; Hb, hemoglobin; RT, radiotherapy; RTCT, concurrent chemoradiation.

* Proportion (%) is to the total cases of early and/or late complications and recurrence.

**Table 3:** Five-Year FCS and Cox Proportional HRs for Combinations of RT and RTCT Used to Treat Locally Advanced (stage IIB-IVA) Cervical Cancers

Type of Treatment	Patients(N = 1,753)	FCS	Unadjusted		Adjusted*	
			HR	95% CI	HR	95% CI
All patients						
RT parameters						
Optimal RT	1,164	61.5	1.0		1.0	
Suboptimal RT	589	56.1	1.21	1.03 to 1.41	1.09	0.92 to 1.29
RT machine						
Linear accelerator	1,215	61.2	1.0		1.0	
Colbalt-60	527	56.2	1.18	1.00 to 1.39	0.96	0.80 to 1.15
RT field						
Two fields	276	41.6	1.0		1.0	
Four fields	1,319	62.1	0.54	0.45 to 0.65	0.55	0.45 to 0.67
> four fields	77	74.9	0.35	0.22 to 0.57	0.36	0.22 to 0.59
RT + CT (cisplatin)	903					
Cisplatin dose categorized beyond 150 mg						
< 150	127	59.5	1.0			
150-199	188	72.6	0.62	0.42 to 0.92		
200-239	195	71.8	0.67	0.46 to 0.97		
≥ 240	247	72.8	0.62	0.43 to 0.89		
Cisplatin dose put together beyond 150 mg						
< 150	127	59.5	1.0			
≥ 150	630	72.4	0.63	0.46 to 0.87		
Dose unknown	146	73.5	0.62	0.41 to 0.94		
RT (808 patients) and RT + CT (cisplatin; 903 patients)	1,711					
RT parameters						
Optimal RT	1,138	61.7	1.0		1.0	
Suboptimal RT	573	55.8	1.23	1.05 to 1.44	1.11	0.95 to 1.30
Cisplatin dose put together beyond 150 mg						
< 150	127	59.5	1.0		1.0	
≥ 150	630	72.4	0.63	0.46 to 0.87	0.64	0.47 to 0.88
Dose unknown	146	73.5	0.62	0.41 to 0.94	0.61	0.40 to 0.93
No chemotherapy	808	47.5	1.46	1.09 to 1.95	1.45	1.09 to 1.94

Abbreviations: FCS, 5-year cumulative survival; HR, hazard ratio; RT, radiotherapy; RTCT, concurrent chemoradiation.

* Adjusted for RT machine and RT field.

**Figure 1:**
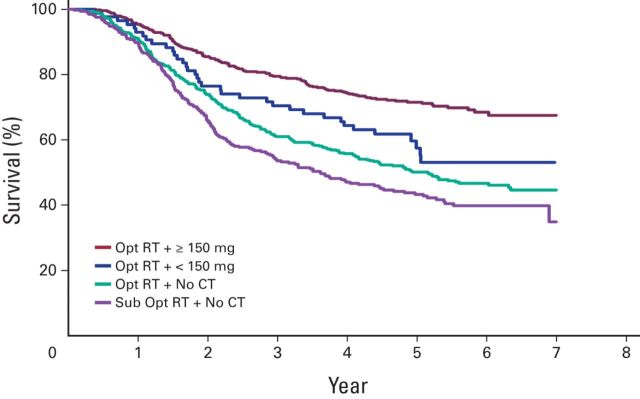
Kaplan-Meier comparative survival graph for combinations of optimal radiotherapy (Opt RT) and at least 150 mg cisplatin as total dose. CT, chemotherapy; Sub Opt RT, suboptimal radiotherapy.

Patients were further regrouped according to combinations of optimal and/or suboptimal RT and total dose of cisplatin (Table 4 and Fig 1). The best survival was seen in patients who received optimal RT and 150 mg or more of cisplatin (FCS, 71.5%) and in those who received suboptimal RT with 150 mg or more of cisplatin (FCS, 76.2%). The shortest survival (FCS, 43.2%) was seen in the group of patients who received suboptimal RT with no CT.

**Table 4:** Five-Year FCS and Cox Proportional HRs for Combinations of Optimal RT and Cisplatin Dose

Type of Treatment	No. of Patients	FCS	Unadjusted		Adjusted*	
			HR	95% CI	HR	95% CI
Optimal RT + ≥ 150 mg cisplatin†	495	71.5	1.0		1.0	
Optimal RT + < 150 mg cisplatin	85	57.4	1.59	1.10 to 2.29	1.71	1.18 to 2.48
Optimal RT + no CT	481	50.1	2.03	1.65 to 2.49	1.89	1.52 to 2.33
Suboptimal RT + no CT	327	43.2	2.49	1.99 to 3.10	2.14	1.67 to 2.73
Suboptimal RT + ≥ 150 mg cisplatin	135	76.2	0.79	0.53 to 1.18	0.75	0.50 to 1.12
Suboptimal RT + < 150 mg cisplatin	42	63.9	1.34	0.79 to 2.28	1.50	0.85 to 2.64
Suboptimal RT + no CT†	327	43.2	1.0		1.0	
Optimal RT + no CT	481	50.1	0.81	0.67 to 0.99	0.88	0.72 to 1.08
Optimal RT + ≥ 150 mg cisplatin	495	71.5	0.40	0.32 to 0.50	0.47	0.37 to 0.60
Optimal RT + < 150 mg cisplatin	85	57.4	0.64	0.45 to 0.92	0.80	0.55 to 1.17
Suboptimal RT + ≥ 150 mg cisplatin	135	76.2	0.32	0.22 to 0.47	0.35	0.24 to 0.52
Suboptimal RT + < 150 mg cisplatin	42	63.9	0.54	0.32 to 0.91	0.70	0.40 to 1.23

Abbreviations: CT, chemotherapy; FCS, 5-year cumulative survival; HR, hazard ratio; RT, radiotherapy.

* Adjusted for RT machine and RT field.

† Reference for comparison.

Comorbidities and performance status were not significantly different (*P* = .25) in the group of patients who received RT alone compared with those who received RTCT. A smaller proportion of elderly patients (age ≥ 65 years) received RTCT compared with those younger than age 65 years. However, there was no statistically significant difference in survival.

Disease-free survival was 45.3% with RT and 69.1% with RTCT (HR, 0.48; 95% CI, 0.41 to 0.56).The relative proportion of early and/or late complications and recurrence was similar in the two groups (Table 2). The number of patients who reported complications increased with increasing dosage of cisplatin (10.2% for patients receiving < 150 mg, 24.8% for 150 to 199 mg, 23.5% for 200 to 239 mg, and 39.8% for ≥ 240 mg). Although an increased complication rate was observed with larger dose, there was no correlation between mortality and dose of cisplatin. The complications recorded were mainly parametrial fibrosis followed by hematologic, GI, and renal complications and skin reactions. These were largely comparable in the two treatment groups.

**Figure 2:**
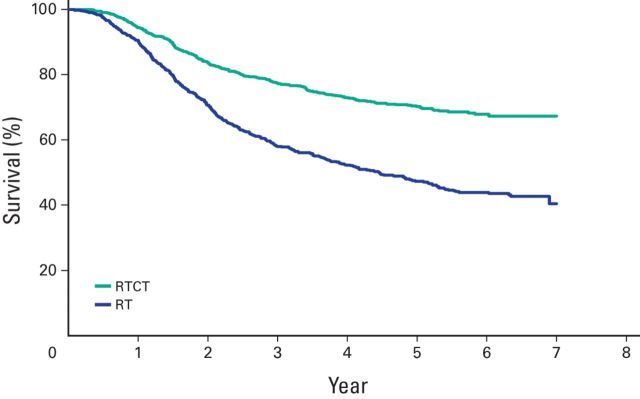
Kaplan-Meier comparative survival graph for patients who received radiotherapy (RT) alone and those who received radiotherapy and chemotherapy (RTCT).

Table 5 compares survival in our study with that in other key publications. The major differences between this study and the others are the sample size (substantially higher in this study) and the study setting (ie, this is the only study from a developing country). Otherwise, the survival benefit of RTCT over RT is comparable. Figure 2 depicts the Kaplan-Meier comparative survival curves for patients who received RT alone and those who received RTCT.

**Table 5:** Comparison of Survival Rates With Relevant Publications

Reference	Study Type	FIGO Stage	Treatment	No. of Patients	5-Year OS (months)
Morris et al^22^	Randomized clinical trial	IB-IVA	RT	195	58
			RTCT	195	73
Eifel et al^23^	Randomized clinical trial	IB-IVA	RT	195	52
			RTCT	195	73
Fujiwara et al^24^	Retrospective	IB2-IVA	RTCT*	52	78
Robert et al^25^	Randomized clinical trial	IB2-IVA	RT	82	56†
			RTCT‡	78	72†
		IB-IVA	RT	1,061	54
This study	Observational		RTCT	1,183	72
		IB2-IVA	RT	971	52
			RTCT	1,145	72

Abbreviations: CT, chemotherapy; FIGO, International Federation of Gynecology and Obstetrics; OS, overall survival; RT, radiotherapy; RTCT, concurrent chemoradiotherapy.

* Nedaplatin-based RTCT.

† Four-year survival.

‡ Chemotherapy with mitomycin.

## DISCUSSION

The survival benefit of RTCT over RT alone was reported in clinical trials in 1999.^22–27^ Since then, and with the alert issued by the National Cancer Institute,^28^ several studies have validated the improved disease-free survival and overall survival of RTCT over RT especially in locally advanced cervical cancer. These studies include updates, systematic reviews, and meta-analyses.^19,23,29^ A recent Chinese study^30^ reported a 5-year overall response rate of 67% in the RTCT arm and 53% in the RT arm. Singh et al^31^ reported less morbidity and mortality with neoadjuvant CT. There have been some contradictory reports.^14,32^ But the authors^14^ concluded that bulky tumors, poor nutritional status, and small sample size could have contributed to differences in outcome not being observed.

The current focus of clinical research has shifted to determining the efficacy of other drugs and examining aspects of tolerance, toxicity, and effectiveness of a lower dose of cisplatin. Ushijima et al^33^ reported a favorable response with an average total dose of 200 mg of cisplatin. We also did not observe significant differences in survival with total cisplatin doses of more than 150 mg. The cisplatin dose compared with RT dose^17^ requires further investigation, at least in the Indian context wherein nutritional status, immunity, and comorbid conditions could play a role.

When the analysis was performed by using optimal and/or suboptimal RT and 150 mg total dose of cisplatin, we found that even patients who had received suboptimal RT but with a total dose of cisplatin of 150 mg or more did as well as or better than those who received optimal RT, suggesting that cisplatin is more important than optimized RT.

Improved survival with RTCT over RT in early-stage (IA2-IIA) cervical cancer has been reported.^34,35^ Surgery and not RTCT had an impact on survival in this study of early-stage cancer.

Most reports of superior survival with RTCT are from developed regions, although a majority of cervical cancers occur in less developed countries. The applicability of the National Cancer Institute^28^ alert or the extent of its implementation in a developing country such as India, where almost 100,000 new cervical cancers in predominantly advanced stage at diagnosis occur annually, has not been investigated. This Indian multi-institutional study has confirmed the distinctly better survival with RTCT with even lower-than-optimal total dose of cisplatin; at the same time, this study reveals that a large proportion of patients with locally advanced cervical cancers are not being given RTCT. This was not a randomized clinical trial, but it nonetheless provides a picture of POC and POS in cancer treatment in India. Observational studies such as POCSS or patient care evaluation studies have several advantages: they are comprehensive, have been externally validated, have broader criteria for patient inclusion, are unbiased, and provide results in routine clinical settings.^36^

Several factors could contribute to patients not receiving CT, such as the lack of qualified/trained clinical oncologists and lack of awareness about the need for such therapy. The data on comorbid conditions and performance status do not suggest that renal insufficiency is a factor in not administering cisplatin. A majority of the patients in this study underwent treatment in established, well-equipped cancer hospitals in which administering chemotherapeutic agents on an inpatient or outpatient basis is a regular daily affair. The reasons for not administering cisplatin probably do not relate to the medical condition of the patient or the facilities available at a given center. Rather, those reasons probably relate to patients' problems such as cost of treatment, the difficulties involved in repeated hospital visits (including long-distance travel), and a false sense of doing well after initial treatment. Data from HBCRs^37^ show that until the year 2000, less than 10% of patients with locally advanced cervical cancers received RTCT. As of 2011, the percentage was 57.9%, but in a medical college HBCR it was 10%.

### Limitations

Pooled data from different types of institutions (cancer centers, medical colleges, private hospitals) has the advantage of representing the complete spectrum of patients and treatment but the disadvantage of having subjective information on some variables that cannot be adjusted in analysis. Standards of care can vary from center to center. There is no selection bias because all patients who received cancer-directed therapy in their respective institutions have been accounted for, and exclusion criteria are based on scientific logic. However, it is possible that a few patients received additional treatment elsewhere which, because of challenges in clinical follow-up, could not be quantified. There was no regular clinical follow-up per National Comprehensive Cancer Network guidelines.^38^ Therefore, details of toxicity, recurrence, and disease-free survival and/or progression-free survival could not be accurately ascertained.

### Strengths and Opportunities

This study is a foremost example of cancer registration because of its national program that evaluates and provides critical findings that could have an impact on patient care. Dynamic Internet-based data capture, data checks, and analysis had several advantages in ensuring standard quality data. NCDIR, a nonprofit organization with software experts as full-time faculty is unique, and along with its medical and statistical expertise, it has the strength of clinical neutrality, a distinct feature spelled out in its manifesto. The NCDIR research panel on cancer and scientific advisory committee have reputed oncologists from all subdisciplines. This POCCS is now an intramural activity of NCDIR. Thus, all 27 regional cancer centers and many other institutions have joined this study, providing an exceptional opportunity to examine, evaluate, and redesign treatment management in cervical and other cancers. A network of cancer hospitals linked to a central coordinating center with a system for accruing good clinical data through modern electronic information technology is in place.

Cancer of the cervix accounts for 93,786^2^ new cancers in 2014, comprising 17.8% of all organ site cancers in Indian women. In India, 80.9% (75,873) of cancers of the cervix present with stage IIB-IVA disease and would require RTCT as standard treatment. More than half (51.1%) of stage IIB-IVA patients received only RT, and their survival is substantially less than those who received RTCT. Thus, annually 38,771 (51% of 75,873) cancers of the cervix do not get the benefit of RTCT and accordingly have poorer survival.

This number—38,771—is conservative because the results presented here are based on treatment provided in some renowned cancer treatment centers that are well equipped in terms of both facilities and skilled staff. In one participating medical college, the proportion of patients who received RTCT was less than 10%. If that proportion is used, then 90% of 75,873, or 68,286 cancers of the cervix, would not receive RTCT.

Perhaps this scenario in standards of care may be no different in other less developed regions of the world, where the majority^39^ of cervical cancers (84.3%) occur. The global burden for 2012 was 527,600.^1^ There seems to be a need to reiterate the NCI^28^ alert that advised supplementing chemotherapy to radiation for locally advanced cancer of the cervix in the context of the developing world. If governments included cisplatin in the list of essential drugs, that would greatly increase its availability and at an affordable cost.

## Appendix

### Patterns of Cancer Care and Survival Study: Cancer Cervix

The study of patterns of cancer care and survival was coordinated, conducted, and funded by the National Centre for Disease Informatics and Research (NCDIR), which is a permanent institute of the Indian Council of Medical Research (ICMR), the premier medical research body of India and part of the Department of Health Research, Ministry of Health and Family Welfare, Government of India. Three initial workshops to finalize the patient information form were supported by the WHO.

All participating institutions had the study protocol cleared by their respective institutional ethics committees, and patient consent was incorporated into the individual patient medical record. The study proposal/protocol was approved and recommended for release of grants at three different levels: first by the members of the Steering Committee of the National Cancer Registry Programme and then by the Research Area Panel on Cancer and Scientific Advisory Committee of the NCDIR; second by the Scientific Advisory Group of the Non Communicable Disease Division of the ICMR; and third by the Biomedical Research Board of ICMR. Ambakumar Nandakumar, the chief principal investigator of the study, was assisted by his team at NCDIR, by members of the Research Area Panel on Cancer, by directors and their clinical and cancer registry colleagues at the cancer centers and medical colleges, and by the concerned staff at ICMR Headquarters in New Delhi.

The Patterns of Cancer Care and Survival Group consisted of the Project Management group whose members and their affiliations include Ambakumar Nandakumar, Francis Selvaraj Roselind, Krishan Sathishkumar, Dampilla Daniel Vijaykumar, Ankush Jain, and Kondalli Lakshminarayana Sudarshan, NCDIR, Bangalore, India. The Cancer Research Area Panel consisted of the following members and their affiliations: Goura Kishor Rath, Institute Rotary Cancer Hospital, All India Institute of Medical Sciences, New Delhi; Amal Chandra Kataki, Dr. B.B. Borooah Cancer Institute, Guwahati; P. Poonamalle Bapsy, Apollo Hospitals, Bangalore; Prakash C. Gupta, Healis-Sekhsaria Institute of Public Health, Navi Mumbai; Paleth Gangadharan, Amrita Institute of Medical Sciences and Research Centre, Kochi; Ramesh C. Mahajan, Post Graduate Institute of Medical Education and Research, Chandigarh; Manas Nath Bandyopadhyay, Cancer Centre Welfare Home and Research Institute, Kolkata; Kumara Swamy, HealthCare Global-Bangalore Institute of Oncology, Bangalore; Elizabeth Vallikad, St. John's Medical College, Bangalore; and Rudrapatna N. Visweswara, International Medical School and M.S. Ramaiah Medical College, Bangalore (all in India).

Collaborators (institutes) supplying individual patient data (in descending order of the number of patients with cancer on whom information was provided, first for survival studies and then for pattern of care only include the Cancer Institute (WIA), Chennai: V. Shanta, R. Swaminathan, G. Selvaluxmy, R. Rama, P. Shanthi, M.S. Kalyani; Regional Cancer Centre, Thiruvananthapuram: Paul Sebastian, Francis V. James, Aswin Kumar, Aleyamma Mathew, Preethi Sara George; Dr B. Borooah Cancer Institute, Regional Cancer Centre, Guwahati: A.C. Kataki, Debabrata Barmon; Amrita Institute of Medical Sciences, Kochi: D.K. Vijaykumar, M. Dinesh, Anupama Rajanbabu, P. Gangadharan; Assam Medical College, Dibrugarh: K. Adhikari, M.S. Ali, R. Akhtar, S.K. Bhuyan, I. Baruah; Cachar Cancer Hospital and Research Centre, Silchar: Ravi Kannan, Ritesh Tapkire, Gopal Dutta, Amit Das, Gayatree Roy; Tata Memorial Hospital, Mumbai: R.A. Badwe, A.K. D'Cruz, B. Ganesh, S.K. Shrivastava, Rajendra Kerkar, Amita Maheshwari, Umesh Mahantshetty, Reena Engineer, Sudeep Gupta, Arshi Khan, Sushama Saoba, Sharwari Joshi, Mitali Sapkal; Kidwai Memorial Institute of Oncology, Bangalore: M. Vijayakumar, K. Ramachandra Reddy, C. Ramesh, D.J. Jayaram; Mahavir Cancer Sansthan, Patna: J.K. Singh, Manisha Singh, Preeti Jain, Anita Kumari; Post Graduate Institute of Medical Education and Research, Chandigarh: Sushmita Ghoshal, Suresh C. Sharma, F.D. Patel; Government Medical College and Hospital, Nagpur: Shuchita Mundle, Pushpa Iyengar; Rajiv Gandhi Cancer Institute and Research Centre, New Delhi: Sheh Rawat, Anjali K. Pahuja, Anamika Mishra; and ICMR Headquarters, New Delhi: V.M. Katoch, D.K. Shukla, Tanvir Kaur.
